# Clinical outcomes of patients with complicated post-operative course after gastrectomy for cancer: a GIRCG study using the GASTRODATA registry

**DOI:** 10.1007/s13304-022-01318-1

**Published:** 2022-07-05

**Authors:** Gian Luca Baiocchi, Simone Giacopuzzi, Giovanni Vittimberga, Stefano De Pascale, Elisabetta Pastorelli, Roberta Gelmini, Jacopo Viganò, Luigina Graziosi, Alessio Vagliasindi, Fausto Rosa, Francesca Steccanella, Paolo Demartini, Rossella Reddavid, Mattia Berselli, Ugo Elmore, Uberto Fumagalli Romario, Maurizio Degiuli, Paolo Morgagni, Daniele Marrelli, Domenico D’Ugo, Riccardo Rosati, Giovanni De Manzoni

**Affiliations:** 1 Department of Clinical and Experimental Sciences, University of Brescia, Brescia, Italy; 2 Department of Surgery, General and Upper G.I. Surgery Division, University of Verona, Verona, Italy; 3 GB Morgagni-L Pierantoni Surgical Department, Forlì, Italy; 4 Department of Digestive Tract Surgery, IEO, Milan, Italy; 5 Department of Oncological Surgery, University of Modena, Modena, Italy; 6 General Surgery, Department of Surgery, Fondazione IRCCS Policlinico San Matteo, Pavia, Italy; 7 General and Emergency Surgery, University of Perugia, Santa Maria della Misericordia Hospital, Perugia, Italy; 8 UOC General and Emergency Surgery, SSD Emergency Surgery, S. Maria delle Croci Hospital, Ravenna, Italy; 9 Department of General Surgery, Fondazione Policlinico Gemelli, Rome, Italy; 10 Aou San Giovanni di Dio e Ruggi d’Aragona, Salerno, Italy; 11 General, Oncological and Minimally Invasive Surgery, Cà Granda-Niguarda Hospital, Milan, Italy; 12 Department of Oncology, Digestive and Surgical Oncology, University of Torino, and San Luigi University Hospital, Orbassano, Italy; 13 General Surgery Unit, Department of Surgery, ASST Settelaghi, Varese, Italy; 14 Gastrointestinal Surgery, San Raffaele Hospital and San Raffaele Vita-Salute University, Milan, Italy; 15 Department of Medicine, Surgery and Neurosciences, Unit of General Surgery and Surgical Oncology, University of Siena, Siena, Italy; 16 UOC General Surgery, ASST Cremona, Cremona, Italy

**Keywords:** Gastric cancer, Complications, Surgery, Post operative mortality

## Abstract

Gastrectomy for gastric cancer is still performed in Western countries with high morbidity and mortality. Post-operative complications are frequent, and effective diagnosis and treatment of complications is crucial to lower the mortality rates. In 2015, a project was launched by the EGCA with the aim of building an agreement on list and definitions of post-operative complications specific for gastrectomy. In 2018, the platform www.gastrodata.org was launched for collecting cases by utilizing this new complication list. In the present paper, the Italian Research Group for Gastric Cancer endorsed a collection of complicated cases in the period 2015–2019, with the aim of investigating the clinical pictures, diagnostic modalities, and treatment approaches, as well as outcome measures of patients experiencing almost one post-operative complication. Fifteen centers across Italy provided 386 cases with a total of 538 complications (mean 1.4 complication/patient). The most frequent complications were non-surgical infections (gastrointestinal, pulmonary, and urinary) and anastomotic leaks, accounting for 29.2% and 17.3% of complicated patients, with a median Clavien–Dindo score of II and IIIB, respectively. Overall mortality of this series was 12.4%, while mortality of patients with anastomotic leak was 25.4%. The clinical presentation with systemic septic signs, the timing of diagnosis, and the hospital volume were the most relevant factors influencing outcome.

## Introduction

Gastric cancer remains one of the most frequently diagnosed and one of the most deadly cancer in the world [1]. Although several progresses has been made in the field of medical therapies, surgical resection with standardized lymphadenectomy still represents a mandatory step in the therapeutic pathway [2–4]. Radical gastrectomy with lymphadenectomy is a complex operation. Many clinical series report very different results in terms of post-operative complications, morbidity, and mortality between eastern and western centers. In-hospital and 30-day mortality rates are less than 1% [5, 6] and more than 5% in East and West, respectively [7, 8]. On the other hand, morbidity rates are reported in a wider range—from 10 to 40% [9, 10]—mainly due to a lack of a standardized reporting system.

In 2015, the project “Complications after gastrectomy for cancer. European perspective” was launched and endorsed by the European chapter of the International Gastric Cancer Association. A group of 31 referral centers from 13 European countries was involved in the Gastrectomy Complications Consensus Group (GCCG), and after a Delphi consensus process, an agreed list including 27 perioperative complications associated with gastrectomy for cancer was developed and published [11]. In 2019, the online platform www.gastrodata.org was launched, providing a tool for clinical, oncological, and surgical data collection. The incidence, grading, and relevant features of complications and outcomes were recorded. Two main studies were performed: a retrospective study comprising all consecutive resections for gastric cancer performed at participating centers in 2017 and 2018 [12], and an observational prospective study focused on interventions performed in 2019–2021 (ongoing). At 31/10/2021, the www.gastrodata.org platform comprises 2531 cases of patients who underwent radical gastrectomy and lymphadenectomy for gastric cancer.

In 2020, during the annual meeting of the Italian Research Group for Gastric Cancer (GIRCG), a study protocol was proposed, entitled “Clinical outcomes of patients with complicated post-operative course after gastrectomy for cancer”. The aim of the study was a deep analysis of post-operative courses when a complication occurs, with special reference to the diagnostic times, the treatment modalities and to the clinical outcomes. All Italian centers participating to GIRC were invited to take part to this study.

## Methods

### Participating centers

The study group consists of 15 Italian centers, which entered data using the standardized gastrodata platform. The centers and the Principal Investigators involved in the study are listed below: University of Brescia (Baiocchi GL), University of Verona (Giacopuzzi S), GB Morgagni-Pierantoni Hospital Forlì (Morgagni P), European Institute of Oncology, Milano (Fumagalli U), Niguarda Hospital, Milano (Demartini P), University of Modena (Deruvo N), S. Matteo Hospital Pavia (Viganò J), University of Perugia (Graziosi L), S. Maria delle Croci Hospital, Ravenna (Vagliasindi A), Catholic University, Gemelli Hospital, Rome (D’ugo D, Rosa F), AOU S. Giovanni Di Dio, Salerno (Steccanella F), University of Siena (Marrelli D), University of Torino (Degiuli M), ASST Settelaghi, Varese (Berselli M), and S. Raffaele Hospital Milano (Rosati R).

### Ethics/study approval

The study was approved by the Institutional Review Boards of the participating centers. The study also meets the guidelines for clinical research required by the institutions with which the authors are affiliated.

### GastroData online platform

The web-based platform www.gastrodata.org was developed by a specialized software firm (www.Fluxedo.com), taking particular attention to the security and anonymity issues. The gastrodata web platform was already approved for data harvesting and collection in 13 European countries in two previous studies [11, 12]. Uniform data collection was allowed by the platform. Each study participant was given personal login credentials to enter data. All data, including center, surgeon, and patient data, were strictly anonymous and managed through secure codes. Each center only had access to its patient data.

Only patients having had a post-operative complication after gastrectomy were included in this study. For these patients, the following data were collected:

A. *Clinical data:* Patient demographics, body mass index (BMI), American Society of Anesthesiologists (ASA) score, Charlson Comorbidity Index, Prognostic Nutritional Index, weight loss, pharmacological therapy at admission, previous supramesocolic surgeries, other major surgeries, Karnofsky Performance Score (KPS), and Eastern Cooperative Oncology Group (ECOG) Performance Status.
B. *Oncological and surgical data:* Preoperative histology (WHO classification), cTNM, diagnostic methods, neoadjuvant chemotherapy, radiotherapy, and chemoradiotherapy, surgical approach, timing, duration, type of procedure, associated resections, lymphadenectomy (as reported by the surgeon), reconstruction, duodenal stump closure, anastomoses, drains, feeding jejunostomy, hyperthermic intraperitoneal chemotherapy, final histology, pTNM or ypTNM, number of harvested and pathological nodes, and Enhanced Recovery After Surgery (ERAS) accomplishment.
C. *Twenty-seven perioperative complications:* One or more complications were recorded for each patient. For each complication, detailed clinical (e.g., post-operative day, presentation, transfer to ICU), radiological (e.g., diagnostic tools), and therapeutic (e.g., type of treatment) data were provided, as well as the complication grading according to the Clavien–Dindo scale [13].
D. *E. F. Outcomes at discharge and at 30 and 90 days post-operatively:* Comprehensive Complications Index (CCI) [14, 15], adjuvant chemotherapy, radiotherapy, chemoradiotherapy, number of hospital re-admissions, number and types of re-interventions (gastrectomy-related or not), escalation of level of care, blood products’ utilization, post-operative hospitalization (days), discharge location, survival, causes of death, KPS, and ECOG Performance Status.

### Study design

This was a retrospective observational study including all consecutive patients undergoing gastrectomy for gastric cancer and having had almost one post-operative complication. The index period was 2015–2019. The primary endpoints of this study were as follows: (i) the most frequently reported complications; (ii) outcome measures: number and type of re-interventions, number of hospital re-admissions, mortality (total and cause-specific) during hospital stay and at 30 days and 90 days post-operatively, blood product utilization, and escalation in level of care; (iii) diagnostic and therapeutic modalities for complicated cases.

### Statistical analysis

Data entry was checked at each center to ensure consistency and avoid biases. Missing entries were checked and required to the participating center by the organizing committee. Some missing information involving pharmacological therapy, KPS, and ECOG Performance Status were allowed. Continuous variables are reported as median and range. Frequencies and percentages are reported for categorical variables. Chi-square test was used for comparison between categorical variables. Statistical analysis was performed using STATA software (version 12, StataCorp LLC, College Station, Texas).

## Results

A total of 927 cases were entered in the gastrodata registry from 15 centers. All patients underwent gastrectomy for cancer in the period 2015–2019. In the present study, 386 patients with R0 resection and with an intra or post-operative complication, as previously defined [11], registered before the 90th post-operative day, were considered for analysis. Out of 386 patients, 248 were males (64.2%) and 138 females (35.8%); mean age was 71.2 years, range 29–94. ASA score > II was recorded in 45.8% of cases and mean BMI was 25.2.

Table 1 shows the clinical data of overall series. Total complications were 538 (mean 1.4 per patient). Mean CCI was 32.4; in more than 1/3 of cases (35.1%), the Clavien–Dindo grading score IIIb or higher was reported. The hospitalization was meanly 23 days long (range 11–162). Surprisingly, only one out of two patients had an escalation of care as a transfer in ICU: 75 cases, 19.4%.

**Table 1:** Complications and outcomes

	Number	Percent	Mean	Median
Complications per patient	1.4	–	–	–
Clavien–Dindo grading of individual complications				
Grade I	66	12.2	–	–
Grade II	172	31.9	–	–
Grade IIIa	121	22.4	–	–
Grade IIIb	76	14.1	–	–
Grade IVa	28	5.2	–	–
Grade IVb	19	3.5	–	–
Grade V	56	10.4	–	–
All	538	–	–	–
Comprehensive Complications Index (CCI)	–	–	32.4	26.2
Post-operative hospitalization (days)	–	–	23.3	17
Patients requiring blood products	110	54.4	–	–
Blood product utilization (number of RBC packages)	–	–	3.9	2.0
Escalation in level of care (mostly to ICU)	75	19.4	–	–

Overall complications are listed in Table 2. Intraoperative complications were rare (1,1% out of 538 total adverse events). The most frequent complications, accounting each for more than 10% of overall complications, were: non-surgical infections (29.2%), anastomotic leak (17.3%), and abnormal fluid from dainage (not related to gastrointestinal leaks, 15.8%). The following complications accounted for 5–10% of adverse events: post-operative bleeding needing urgent transfusions or invasive treatment (13.4%), pancreatic leak (8.8%), duodenal leak (8.0%), and bowel obstruction (7.7%).

**Table 2:** Incidence of complications and median Clavien–Dindo grading score in 386 patients with almost one post-operative complication

		Adverse events (num)	Adverse events (%)	Clavien–Dindo score (median)
Intraoperative				
18	Unintended intraoperative damage to major vessels and/or organs requiring reconstruction or resection	4	1.0	–
20	Intraoperative bleeding requiring urgent transfusion	2	0.5	–
22	Unexpected medical conditions interrupting or changing the planned procedure	0	0.0	–
Post-operative general				
1	Non-surgical infections	113	29.2	II
9	Pleural effusion requiring drainage	18	4.6	IIIa
10	Pulmonary embolism	14	3.6	IVa
11	Respiratory failure requiring re-intubation	13	3.3	IVa/IVb
13	Acute renal insufficiency/renal failure requiring CVVH/ dialysis	10	2.6	II
14	Need for prolonged intubation (> 24 h after surgery)	8	2.1	III
15	Cardiac dysrhythmia requiring invasive treatment	7	1.8	Unknown
16	Acute myocardial failure with acute pulmonary edema	6	1.5	V
17	Acute liver dysfunction (Child–Pugh > 8 for 48 + hours)	5	1.3	II
19	Pneumothorax requiring treatment	3	0.7	IIIa
20	Need for CPR	2	0.5	IVa/V
20	Myocardial infarction	2	0.5	II/IIIa
21	Need for tracheostomy	1	0.3	V
22	Stroke causing patient's permanent deficit	0	0.0	–
Post-operative surgical				
2	Anastomotic leak	67	17.3	IIIb
3	Other post-operative abnormal fluid from drainage, abdominal collections without gastrointestinal leak(s)	61	15.8	II
4	Post-operative bleeding requiring invasive treatment	52	13.4	IIIb
5	Post-operative pancreatic fistula	34	8.8	II
6	Duodenal leak	31	8.0	IIIa
7	Post-operative bowel obstruction	30	7.7	IIIa
8	Other major complications requiring re-intervention or other invasive procedures	21	5.4	IIIb
12	Post-operative bowel perforation or necrosis	12	3.1	IVb
12	Delayed gastric emptying (by 10th post-operative day)	12	3.1	I/IIIa
13	Post-operative pancreatitis	10	2.6	II
Total number of adverse events		538	–	–

Complications are numbered as per incidence

Table 3 shows the clinical outcomes. The most important outcome is mortality, which is reported at discharge, 30 and 90 pod. Overall mortality of the series was 12.5%, including 8% of deaths due to complications. Reinterventions were needed in 27.2% of cases.

**Table 3:** Mortality and causes of death at discharge, after 30 and 90 pod

Mortality		
Cause of death	Num	%
At discharge		
Related to the surgical procedure	26	6.7
Unrelated to the tumor and the surgical procedure	12	3.1
Total	38	9.8
At 30 days		
Related to the surgical procedure	25	6.5
Unrelated to the tumor and the surgical procedure	11	2.8
Total	36	9.3
At 90 days		
Related to the surgical procedure	31	8.0
Unrelated to the tumor and the surgical procedure	13	3.4
Tumor progression	4	1.0
Total	48	12.4

The most frequent complication was anastomostic leak. Clinical and radiological data and grading are reported in Table 4. About 1 patient out of 4 with anastomotic leak finally died (25.4%); 37.4% of them graded Clavien–Dindo IIIb or more. A re-intervention was necessary in 44.8% of cases, endoscopic treatment was done in 61.2%, and a percutanours drainage in 23.9% of cases. Post-operative stay was as long as 40 days, and mean CCI was 55.5.

**Table 4:** Clinical and radiological data of patients having anastomotic leak

	Num	%	Median	Mean	Range
Patients with anastomotic leak	67	17.3	–	–	–
Grade I	1	1.5	–	–	–
Grade II	10	14.9	–	–	–
Grado IIIa	14	20.9	–	–	–
Grado IIIb	19	28.4			
Grado Iva–IVb	6	9.0	–	–	–
Grade V	17	25.4	–	–	–
Pod	–	–	5	6.8	1–27
Anastomosis					
Esophago-jejuno	51	76.1	–	–	–
Gastro-jejuno	12	17.9	–	–	–
Jejuno-jejuno	4	6.0	–	–	–
Surgical procedure					
Extended total gastrectomy	6	9.0	–	–	–
Total gastrectomy	42	62.7	–	–	–
Subtotal gastrectomy	17	25.4	–	–	–
Proximal	2	3.0	–	–	–
Presentation (multiple options allowed)					
Biliary/enteric content in abdominal drains	46	68.6	–	–	–
Peritonitis	29	43.3	–	–	–
Asymptomatic/radiological findings	4	6.0	–	–	–
Pleural empyema	1	1.5	–	–	–
Methylene blue in drainage after oral administration	2	3	–	–	–
Systemic signs (sepsis/septic shock)	38	56.7	–	–	–
Diagnosis method (multiple options allowed)					
CT scan (iv and/or per os contrast medium)	51	76.1	–	–	–
Clinical	48	71,6	–	–	–
Oral contrast medium X-ray	19	28,4	–	–	–
Endoscopy	15	22,4	–	–	–
Laparotomy	8	11,9	–	–	–
Mean leak volume during the first 5 days after surgery					
< 200 ml/day	43	64.2	–	–	–
201–500 ml/day	6	9.0	–	–	–
Unknown	18	26.9	–	–	–
Treatment (multiple options allowed)					
Surgical	30	44.8	–	–	–
Endoscopic	41	61.2	–	–	–
Percutaneous drainage	16	23.9	–	–	–
Nasogastric tube	21	31.3	–	–	–
Feeding jejunostomy	8	11.9	–	–	–
Fasting and parenteral nutrition	25	37.3	–	–	–
No invasive treatment (antibiotics are included)	13	19.4	–	–	–
Outcome					
Complete leak closure	47	70.1	–	–	–
No leak closure	14	20.9	–	–	–
Unknown	6	9.0	–	–	–
Leak duration (days)	–	–	20.0	34.2	3–175
Post-operative hospitalization (days)	–	–	33.0	40.0	10–120
CCI	–	–	42.5	55.5	20.9–100
Dead patients due to this complication	17	25.4	–	–	–

Table 5 shows the comparison of mortality with some clinical findings in patients with anastomotic leak. In particular, two features appeared related to mortality: the clinical presentation of anastomotic leak in terms of systemic signs of sepsis or septic shock and the timing of diagnosis with respect to the symptoms (or change in drain) onset. Hospital volume, taking 500 beds as cut-off, was not significantly related to mortaly (*p* = 0.08), even a trend is evident; this may be due to the small number of cases.

**Table 5:** Comparison of mortality with clinical presentation, diagnostic time and hospital volume in patients with anastomotic leak

	Death	Survive	*P*
Systemic septic signs			
Yes	13	24	0.041
No	4	26	
Diagnostic time after symptoms onset			
< 24 h	2	22	0.016
> 24 h	15	28	
Hospital volume			
> 500 beds	4	24	0.087
< 500 beds	13	27	

## Discussion

The present paper reports the first study performed by utilizing the data of the GASTRODATA registry after his presentation to the scientific community. This registry contains a big number of data, including demographic, surgical, pathological, radiological, and prognostic ones, and may be the basis for a potentially infinite number of sub-studies.

The most important feature of the GASTRODATA registry is the commonly agreed language, born after a long and intense multicentric work. The list of 27 complications was published in 2018 by the working group appointed by EGCA in 2015 [11]; the same group launched thereafter the www.gastrodata website.

The Italian Research Group for Gastric Cancer would like to investigate only patients having experienced a complication in the post-operative course. The aim of the study is a clinical analysis of post-operative paths, in terms of most frequently represented complication(s), diagnostic modalities, therapeutic approach, and final outcome. This is a typical western population, in which 70-year-old, stout patients, with various comorbidities were predominant. At least half of patients had lost weight, half of them underwent neoadjuvant chemotherapy, and 60% had T3/T4 cancer, while only 20% of patients had early gastric cancers; the proximal localization of the tumor was reported in 60% of cases, and the majority of them underwent open surgery and D2 lymphadenectomy [12].

The main messages of this series were the following.

1. The most frequently reported complications were non-surgical infections (29.2%) and anastomotic leak (17.3%). The incidence of these complications has been reported by the previous analysis of the whole dataset to be 23% and 9.8% [12], respectively. Median CD score was II and IIIb, respectively. Adding duodenal leaks to anastomotic leaks accounted for another 8.0%, for a total of 25.3% over the global series of patients with complicated post-operative course. Non-surgical infections means the presence of urinary, pulmonary, and gastrointestinal infection signs or symptoms associated with microbiological isolations. The most important and clinically relevant were pulmonary infection, sometimes needing pleural drainage and re-intubation. These figures are similar to those recently reported by Gertsen et al. from the DUCA registry [7]. There is no obvious line of action regarding these complications. Some experts suggest to collect preoperative swabs (mouth, stool, urine, and sputum) from patients undergoing surgery as a way to address the eventual post-operative infection therapy. Respiratory complications may be attenuated by boosting the minimally invasive approach, imposing abstinence of smoking, providing pain management and ERAS programs, and planning respiratory pre-habilitation [16]. Regarding anastomotic leaks, 76% of them were at the esophago-jejunal anastomosis, and occurred after total or extended total gastrectomy. A portion of leaks may be due to patient-related factor. However, a portion of leaks may be linked to the employed surgical technique [17], calling for action regarding the improvement in the learning of surgical techniques [18–20].
2. The diagnostic path of anastomotic leaks deserves to be deeply analyzed. Contrast-enhanced CT scan was the most used method (76.1%), while contrast swallow was reported only in 28.4% of cases and endoscopy in only 22.4% of cases. However, a clinical diagnosis (systemic signs of sepsis or septic shock, drain content, and methylene blue injection) was done in three out of four patients. The most important point is related to diagnostic timing. As shown in Table 5, a delay in recognition of the complication significantly translates into a worsening of mortality rate. Indeed, two different paths has been recorded: in many cases patients having an anastomotic leak which had a very heavy impact on outcomes, some suspicious clinical signs would have been recorded some hours or days before; the consequence was a delay in treatment, both systemic (antibiotics, rest, iv feeding) and local. On the contrary, the majority of patients finally rescued had a prompt diagnosis and immediate treatment (Table 5).
3. In the present paper, it was not possible to compare the clinical outcomes to the volume of gastric cancer surgery of each center; on the other hand, data about hospitals were available; thus, a cut-off of 500 beds was chosen to identify high-volume hospitals. The comparison between hospital volume and outcomes showed a clear tendency to significancy (*p* = 0.08). The analysis of treatment of anastomotic leak was difficult, because each patient has a clinical course with specificities related to general conditions, timing of diagnosis, facilities availability. For instance, some cases were managed during weekend, and some hospital does not have endoscopy and interventional radiology available in these days [21]. Thus, we could not identify the best treatment modality of anastomotic leak. Patients undergoing surgery had the worse outcome, but these data are biased, because surgery was employed only after failure or unavailability of endoscopic and radiological treatments; these cases are obviously the most difficult. Moreover, an effective treatment of esophago-jejunal leak (the eso-sponge system) was not available in the first years of this series.
4. Clinical outcomes of patients with complicate course were impressively heavy: in the whole series, mortality after 90 days was 12.4%; in the group of patients with anastomotic leak, mortality was 25.4%. Mean hospital stay was very long: 23.3 days in the whole series, 40 in the patients with anastomotic leak.

The present study does have limitations. Being a retrospective and multicentric study, quality of surgery and quality of complication management could not be assured across the participating centers [22, 23]. Moreover, some data regarding the crucial clinical decisions are missing; thus, the precise clinical path of each patient could not be clearly reconstructed.

In conclusion, radical surgery for gastric cancer still involves high morbidity and mortality rates. Understanding the factors associated with these higher mortality and morbidity rates is critical [24–27]. The list of the most frequent complications presented in this study can help address this issue.
